# Colombian centenarians: A Geohealth approach to longevity study

**DOI:** 10.1371/journal.pone.0311690

**Published:** 2025-02-21

**Authors:** Clímaco de Jesús Pérez-Molina, Flor Elena Chavarro-Bermeo, Luis-Fernando Gutiérrez-Fernández, Santiago Galvis-Villamizar, Wanderley Augusto Arias Ortiz, Laura Cabezas-Pinzón, Carlos-Felipe Escobar-Roa

**Affiliations:** 1 Doctorate in Public Health, School of Medicine, Complexity and Health Research Group, Universidad El Bosque, Bogotá D.C., Colombia; 2 School of Medicine, Community Medicine and Collective Health Research Group, Universidad El Bosque, Bogotá D.C., Colombia; 3 Vicerrectoría de Investigación, Grupo de Investigación en Saneamiento Ecológico, Salud y Medio Ambiente, Instituto de Salud y Medio Ambiente, Universidad El Bosque, Bogotá D.C., Colombia; 4 Innovation Center—iEx Hub (Innovation and Extension Hub), Universidad El Bosque University, Bogotá D.C., Colombia; University of Palermo, ITALY

## Abstract

**Introduction:**

Population longevity is a global phenomenon influenced by various factors including social, economic transitions, and medical advancements. The study focused on the population over 95 years old, adopting an approach that integrates data from the 2018 Census and geospatial analysis techniques.

**Methods:**

An ecological study was conducted using anonymized microdata from the 2018 National Population and Housing Census (CNPV). Geographic analysis, choropleth maps, and Kernel density estimation were employed to identify clusters of individuals aged over 95 years.

**Results:**

The study identified 43,427 individuals aged 95 years or older in Colombia, with concentrations observed in departments such as Antioquia and Bogotá. Analysis by department and municipality revealed variations in rates and sex distribution. Kernel density analysis highlighted clusters in the Valle de Tenza area and other regions.

**Conclusion:**

This study sheds light on the geographical distribution of centenarians in Colombia, emphasizing clusters in certain regions. More research is needed to understand the individual and contextual factors underlying successful aging in Colombia and to inform policies to improve the quality of life of older populations.

## Introduction

Population longevity is a global phenomenon that has gained particular importance in recent decades, driven by social, demographic, economic transitions, and advances in medical care. These elements have contributed to a significant increase in human life expectancy [1, 2]. Before the second half of the 19th century, global life expectancy at birth was less than 30 years, with high mortality during the early years of life; however, by 1950, the global average had increased to approximately 45.7 years, and by 2020, it reached 73 years [3]. According to WHO projections, by the year 2050, the population over 60 years old will represent 22% of the total, with 80% of this population residing in low and middle-income countries [4].

Successful aging, understood as the ability to reach advanced ages, has captured the attention of the scientific community to understand the factors contributing to it [5]. Similarly, Colombia is no exception, and analysis of data from the 2018 National Census revealed a significant presence of individuals over 95 years old [6]. This phenomenon requires studies to understand the conditions of their life cycle, such as increased accumulated experience, promotion of social cohesion, and intergenerational support, which are relevant aspects for the well-being of these communities.

The pathological approach has predominated so far, with few references to the phenomenon as a complex and multidimensional process, whose comprehensive understanding requires approaches considering the dynamic interaction between the organism, the environment, and the socio-cultural circumstances of communities and individuals [7, 8]. Recent literature has explored various factors underlying extreme longevity, including genetic, environmental, nutritional, and lifestyle influences [9, 10]. Studies on Blue Zones, regions with high concentrations of centenarians, have identified common lifestyle characteristics contributing to longevity, such as plant-based diets, regular physical activity, strong social networks, and low-stress levels [11, 12]. In this sense, exploring the geographic, social, and environmental conditions that favor longevity becomes a relevant task to address challenges and opportunities for this population today, as such analysis can provide information to promote comprehensive and effective approaches [13–15].

Colombia, like most countries in the world, is experiencing a rapid aging process of its population [16, 17]. According to DANE figures, by the year 2021, 13.9% of the population was aged 60 or older; according to projections for 2050, this figure could increase to 25%. For the 2005 population census, the number of centenarians was 3,165 people reaching 12,226 by 2018 [4, 18–22].

The work of Rosselli 2017 focused on the geographical description analysis of the centenarian population at the departmental and municipal levels [23, 24]. While other studies delved into clinical, epidemiological, and demographic analysis of cohorts of individuals over 95 years old [25, 26]. More recently, other research explored the sociodemographic characteristics associated with exceptional longevity, using available census data [26, 27]. In contrast to these studies, which address conventional political-administrative divisions without establishing relationships between identified municipalities, this work adopted an approach that integrated and analyzed data from the 2018 Census, from a Geosalud perspective [5]. Population rates over 95 years old per municipality (projected for 2024) were used, applying a criterion of geographical proximity to group and analyze available data; thus, the existence of specific territories where such population was concentrated was identified, revealing patterns and trends not addressed in previous analyses. This was achieved through tools such as Kernel heat maps, to visualize the main clusters of exceptional longevity.

## Methods

An ecological study with a descriptive scope was conducted using secondary data sources. Anonymized microdata from individuals in the 2018 National Population and Housing Census (CNPV) were obtained from the National Data Archive (ANDA) of the National Administrative Department of Statistics (DANE).

The databases of the departments were concatenated and filtered for the population over 95 years old. Variables such as department, municipality of residence, age group, sex, and the question of "place of residence of the citizen five years ago" were considering assessing whether residence in the municipality was permanent or transitory.

For analysis, the absolute and relative frequency of individuals was estimated, followed by calculation of rates per 10,000 inhabitants, following the methodology proposed by Roselli D et al (2017) [24]. The rate was calculated for the general population and by sex. Male-female ratios (masculinity) per 100 women were also estimated.

In the analysis, geographical coordinates (latitude and longitude) were obtained for departments and municipalities from the National Geostatistical Framework (MGN) of DANE. Subsequently, choropleth, density, and heat maps were created. Kernel Density method was used to identify density concentrations, which employs kernel density estimation to create a density raster from a vector point layer.

In data preparation, concatenation, and filtering, Pandas and Glob libraries in Python were used via a Jupyter Notebook in the Anaconda 3 environment. Descriptive and statistical analysis utilized SPSS version 26, Microsoft Excel 365 for rate calculation, and creation of choropleth maps using Bing. Geographic analysis was performed using ArcGIS Pro.

## Results

**Fig 1:**
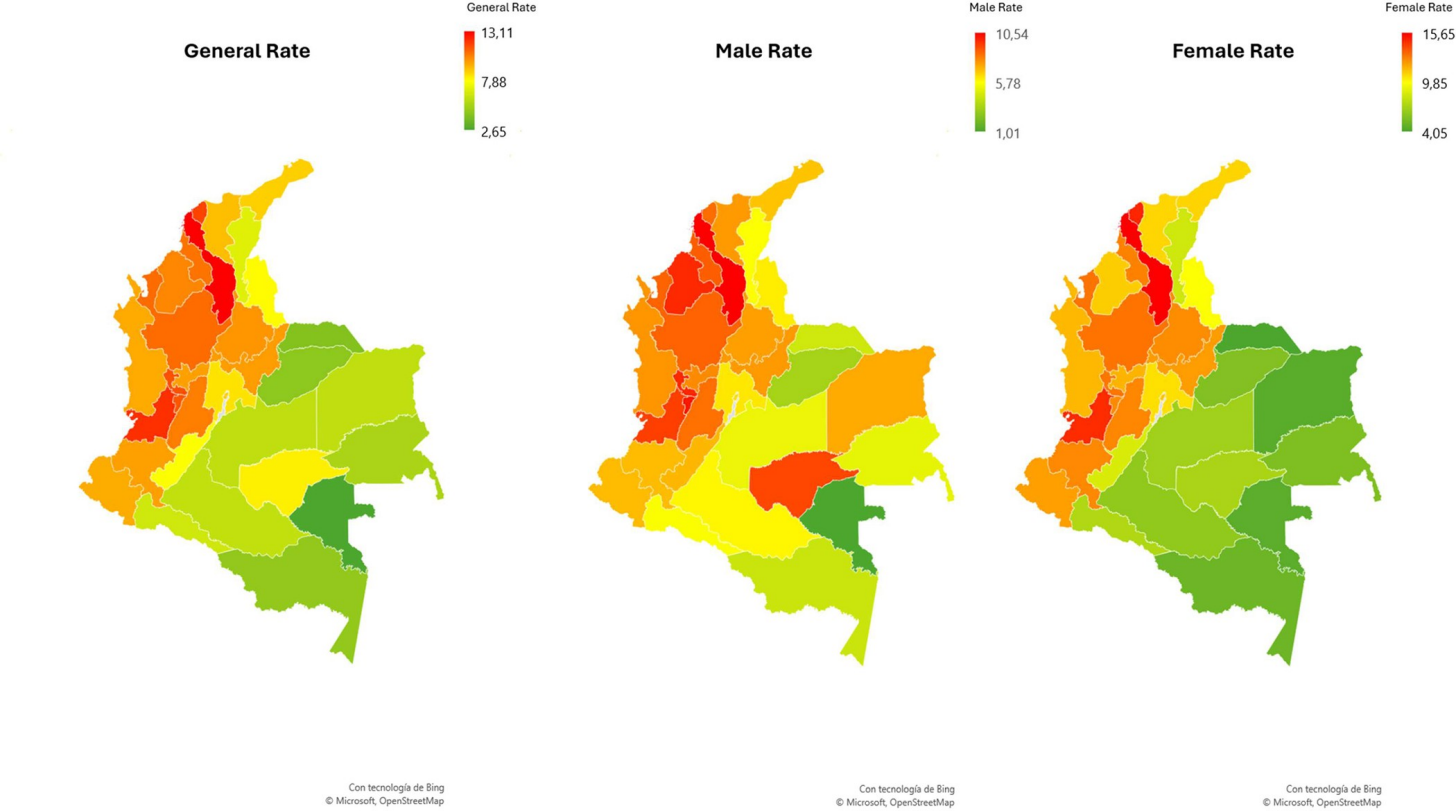
Distribution by departmental rates by sex of the population over or equal to 95 years of age per 10,000 inhabitants in Colombia, according to the CNPV 2018.

According to the 2018 National Census, a total of 43,427 individuals aged 95 years or older were identified. Among these, the highest proportions were found in the departments of Antioquia, with 14.99% (n = 6,509), and Bogotá, with 14.73% (n = 6,397). Conversely, the lowest proportions were observed in the departments of Guainía, with 0.06% (n = 24), Amazonas, with 0.07% (n = 31), and San Andrés, Providencia, and Santa Catalina, with 0.08% (n = 36) (See Fig 1).

Regarding the distribution of individuals aged over 95 years per 10,000 inhabitants, it was found that the department with the highest rate was Bolívar, with 13.11, followed by Valle del Cauca, with 12.13. On the other hand, Vaupés was the department with the lowest rate, with 2.65. In terms of masculinity ratio, Vichada department had the highest proportion, with 187.50 men per 100 women, while Vaupés recorded the lowest, with 25.00 men per 100 women (see Table 1).

**Table 1:** Distribution by department of the population aged 95 years or older in Colombia, according to the CNPV, 2018.

Department	Population (≥95 years)				Rate per-10.000 inhabitants				Rate
	*T*	*M*	*W*	*T*		*W*	*M*	*M*:*W*	
Antioquia	6509	2500	4009	10,89		8,66	12,98	62,36	
Atlántico	2746	968	1778	11,72		8,49	14,79	54,44	
Bogotá	6397	2199	4198	8,91		6,40	11,20	52,38	
Bolívar	2504	999	1505	13,11		10,54	15,65	66,38	
Boyacá	1124	428	696	9,90		7,66	12,07	61,49	
Caldas	904	355	549	9,79		7,93	11,53	64,66	
Caquetá	219	108	111	6,09		5,92	6,26	97,30	
Cauca	1220	433	787	9,81		7,03	12,54	55,02	
Cesar	770	311	459	7,01		5,72	8,27	67,76	
Córdoba	1611	755	856	10,36		9,75	10,96	88,20	
Cundinamarca	2354	859	1495	8,43		6,23	10,58	57,46	
Chocó	440	175	265	9,62		7,74	11,45	66,04	
Huila	794	358	436	7,86		7,11	8,62	82,11	
La Guajira	732	277	455	8,87		6,85	10,80	60,88	
Magdalena	1164	484	680	9,21		7,65	10,77	71,18	
Meta	550	244	306	5,98		5,25	6,74	79,74	
Nariño	1281	461	820	9,59		7,05	12,03	56,22	
Norte de Santander	1074	406	668	7,97		6,11	9,78	60,78	
Quindío	577	248	329	11,32		10,10	12,46	75,38	
Risaralda	960	393	567	11,43		9,78	12,95	69,31	
Santander	2018	742	1276	10,05		7,54	12,44	58,15	
Sucre	925	381	544	10,71		8,77	12,66	70,04	
Tolima	1293	514	779	10,52		8,44	12,57	65,98	
Valle del Cauca	4596	1682	2914	12,13		9,34	14,65	57,72	
Arauca	102	54	48	4,26		4,46	4,05	112,50	
Casanare	173	65	108	4,55		3,39	5,75	60,19	
Putumayo	183	81	102	6,46		5,67	7,27	79,41	
Archipiélago de San Andrés, Providencia y Santa Catalina	36	10	26	7,45		4,29	10,41	38,46	
Amazonas	31	15	16	4,69		4,36	5,06	93,75	
Guainía	24	12	12	5,40		5,17	5,66	100,00	
Guaviare	60	36	24	8,21		9,21	7,06	150,00	
Vaupés	10	2	8	2,65		1,01	4,47	25,00	
Vichada	46	30	16	6,00		7,37	4,45	187,50	

Calculations of the rates per 10,000 inhabitants.

**Fig 2:**
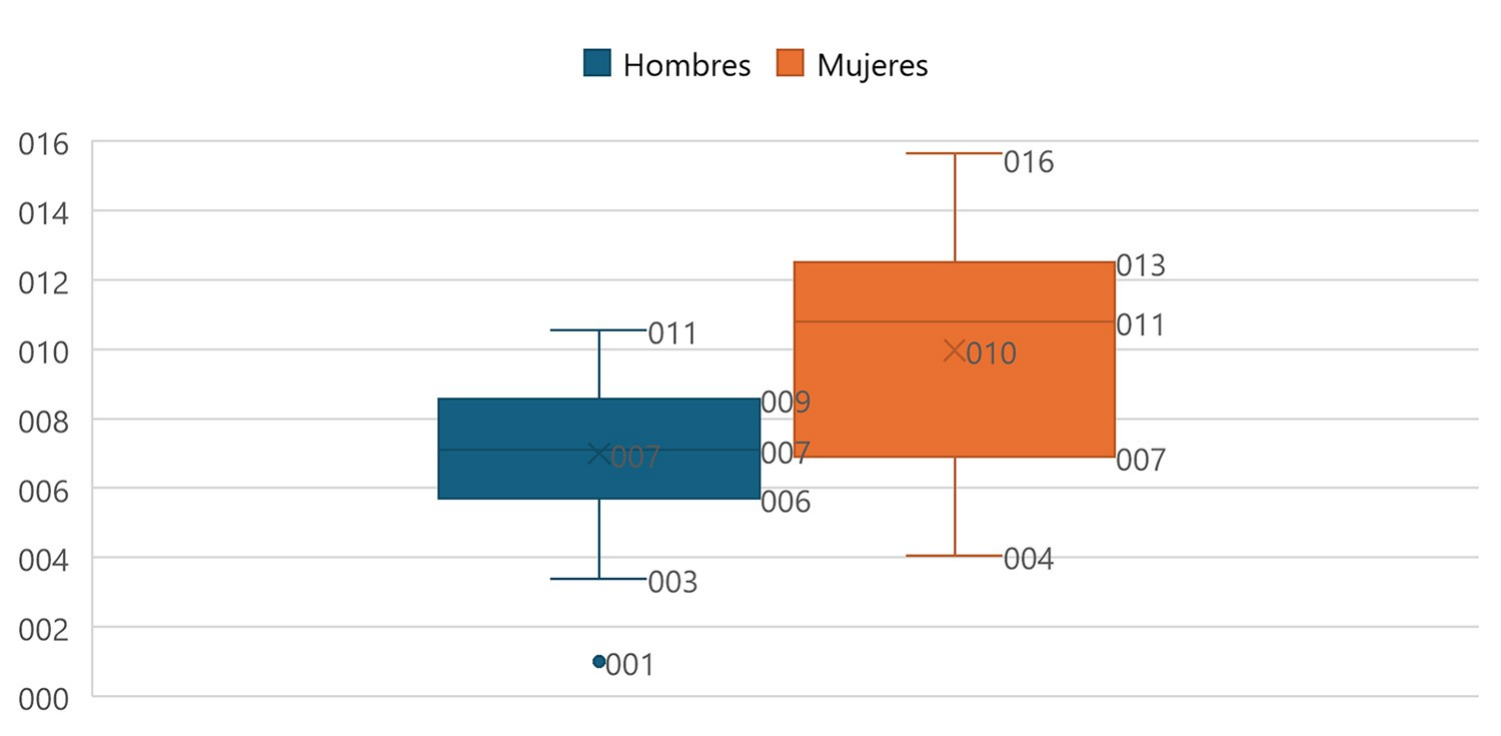
Distribution of departmental rates by sex of the population over or equal to 95 years of age in Colombia, according to the CNPV, 2018.

At the national level, in terms of sex distribution, it was identified that, overall, in the male sex, there is an average rate of 7.00 with a range [1.01–10.54], while, in the female sex, a rate of 9.97 with a range [4.05–15.65] was identified nationwide (See Fig 2).

In the municipality analysis, a national average rate of 10.30 per 10,000 inhabitants was found. The municipalities with the highest representation were La Celia (Risaralda), with a rate of 76.07 (n = 47), followed by Ciudad Bolívar (Antioquia) with a rate of 64.21 (n = 150), and Sutatenza (Boyacá) with a rate of 45.70 (n = 16). On the other hand, the municipalities with the lowest rate were La Montañita (Caquetá) with 0.82 (n = 1) and Cubará (Boyacá) with 0.98 (n = 1), specifying the top 10 municipalities by general rate per 10,000 inhabitants, respectively. In the analysis of origin, regarding their municipality of residence five years ago, in the top ten municipalities, it was found that the proportion varied between [71.43–100%], indicating that in these municipalities, at least three-quarters of the population aged over 95 years have lived in the same place for the last five years (See Table 2).

**Table 2:** Distribution by municipality in the top 10 municipalities by general rate, of the population greater than or equal to 95 in Colombia, according to the CNPV 2018.

Name City	Population (≥95 years)			Rate per-10.000 inhabitants				Rate M: W	% same municipality
	T	M	W		T	W	M		
La Celia (Risaralda)	47	33	14		76,08	101,57	47,80	235,71	89.36
Ciudad Bolívar (Antioquia)	150	99	51		64,21	84,75	43,67	194,12	88.67
Sutatenza (Boyacá)	16	6	10		45,70	35,09	55,83	60,00	81.25
Panqueba (Boyacá)	7	3	4		42,58	37,08	47,90	75,00	71.43
Manta (Cundinamarca)	14	4	10		41,74	23,36	60,90	40,00	92.86
Berbeo (Boyacá)	6	3	3		41,29	40,65	41,96	100,00	100.00
El Piñón (Magdalena)	68	42	26		39,29	46,50	31,42	161,54	100.00
La Victoria (Valle del Cauca)	42	20	22		37,98	37,98	37,98	90,91	90.48
La Capilla (Boyacá)	8	2	6		34,78	17,20	52,77	33,33	87.50
Macanal (Boyacá)	12	4	8		33,63	22,16	45,38	50,00	75.00

Calculations of the rates per 10,000 inhabitants.

**Fig 3:**
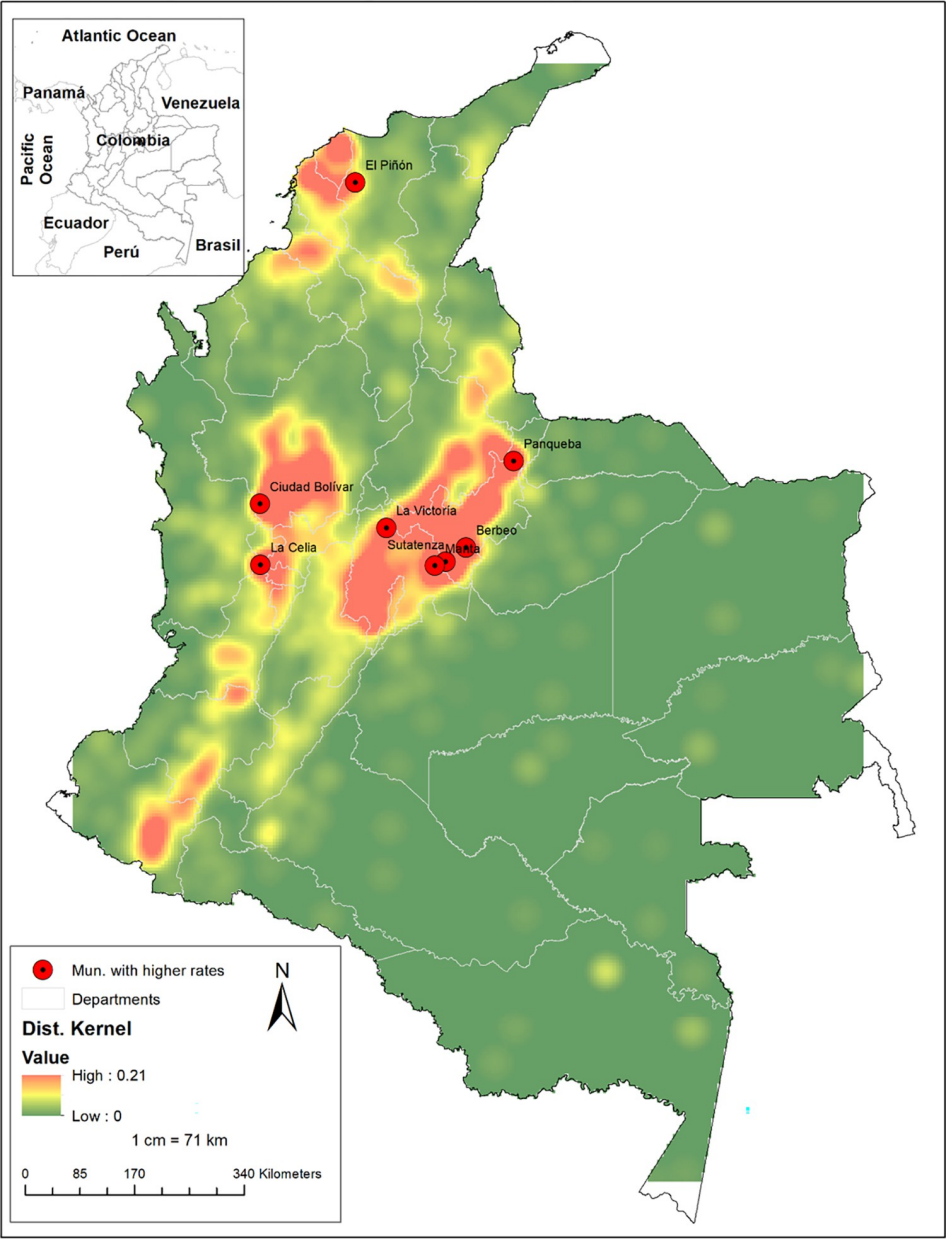
Geographic analysis of centenarian concentrations in Colombia using Kernel Density.

The distribution by sex showed that Chinavita (Boyacá), Almeida (Boyacá), and Chivor (Boyacá) had no male representation. On the other hand, in terms of masculinity ratio, Puerto Gaitán (Meta) recorded the highest ratio, with 10 men per woman, followed by Gualmatán (Nariño) and Sabanas de San Ángel (Magdalena), with 700 men per 100 women each. Forty municipalities were identified without women, and 62 without men aged 95 years or older. Kernel density analysis revealed a geographic concentration in the area near the Valle de Tenza, involving 17 municipalities. Nine of these were among the top 100 municipalities in the country by general rate, and 15 among the top 500 (See Fig 3).

**Fig 4:**
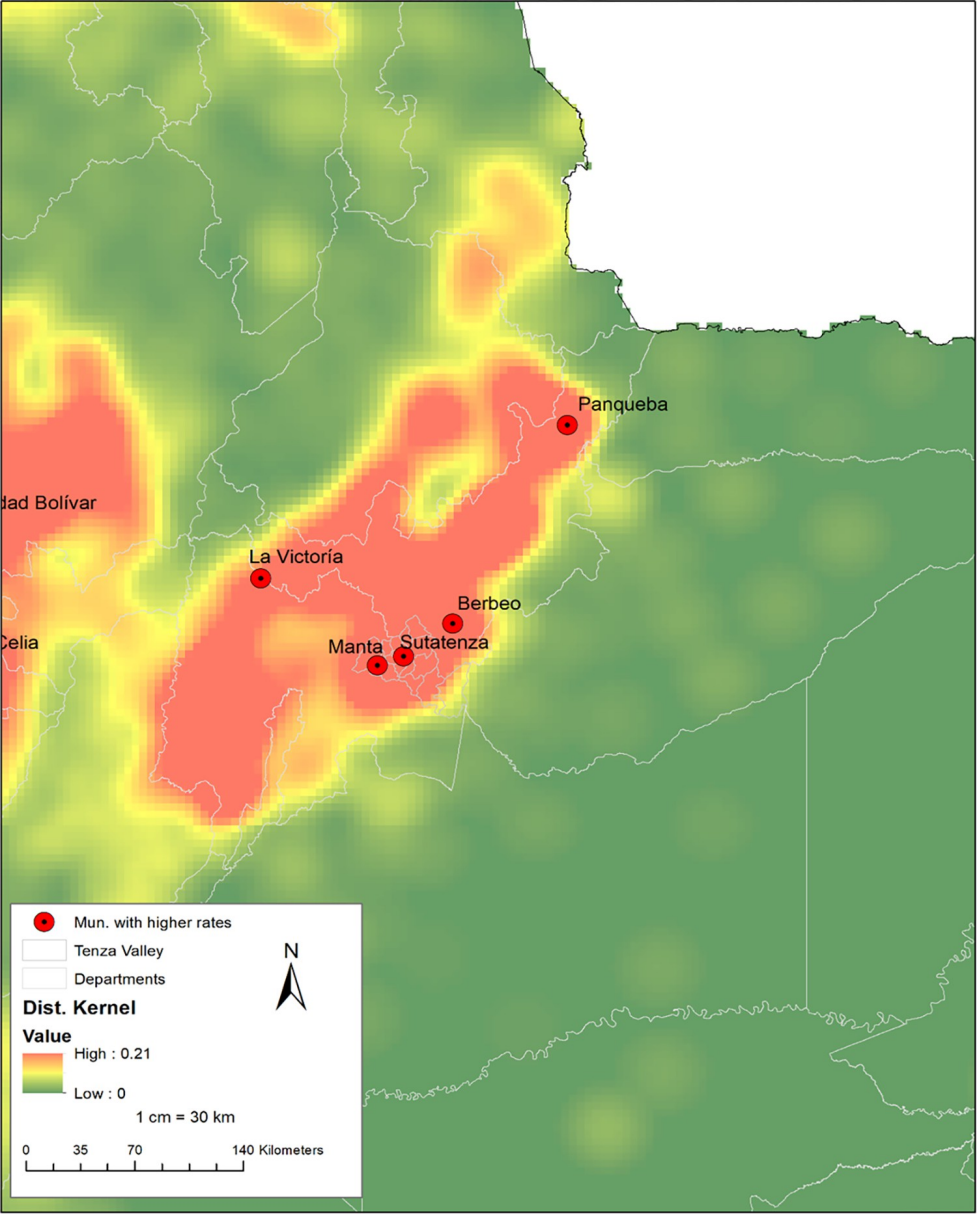
Zoomed-in geographic analysis of centenarian concentrations in the Tenza Valley using Kernel Density.

The general rate in the area near the Valle de Tenza varied between [5.99–45.70], with 10 municipalities above the national average. The general rates of individuals aged over 95 years, for the three municipalities belonging to Cundinamarca (Macheta, Manta, and Tiribita), are above the national average and also above the departmental average 8.43 (Cundinamarca) (See Fig 4).

In the analysis by proportion of inhabitants living in the municipalities of the Tenza Valley in the last five years, it was identified that more than 75.00% lived in the same municipality. However, in the municipality of Tenza (Boyacá) only 57.14% of residents and 66.67% of Pachavita residents (See Table 3).

**Table 3:** Distribution by municipality of the population greater than or equal to 95 in the Tenza Valley, according to the CNPV 2018.

Position	Municipality	Population (≥95 years)				Rate per-10.000 inhabitants		Rate	% Same municipality
		Total	M	W	Total	M	W	M:W	
3	Sutatenza (Boyacá)	16	6	10	45,7	35,09	55,83	60	81,25%
5	Manta (Cundinamarca)	14	4	10	41,74	23,36	60,9	40	92,86%
9	La Capilla (Boyacá)	8	2	6	34,78	17,2	52,77	33,33	87,50%
10	Macanal (Boyacá)	12	4	8	33,63	22,16	45,38	50	75,00%
22	Pachavita (Boyacá)	6	2	4	26,37	17,01	36,4	50	66,67%
30	Chinavita (Boyacá)	7	0	7	24,63	-	48,44	-	100,00%
51	Somondoco (Boyacá)	5	3	2	21,03	25,77	16,49	150	100,00%
64	Tenza (Boyacá)	7	1	6	20,23	5,87	34,17	16,67	57,14%
78	Almeida (Boyacá)	3	0	3	18,96	-	39,89	-	100,00%
121	Garagoa (Boyacá)	24	6	18	16,97	8,97	24,14	33,33	87,50%
141	Santa María (Boyacá)	5	2	3	16,08	12,48	19,91	66,67	100,00%
142	Guateque (Boyacá)	13	6	7	16	15,63	16,34	85,71	100,00%
162	San Luis de Gaceno (Boyacá)	7	5	2	15,38	21,52	8,98	250	100,00%
305	Tibirita (Cundinamarca)	3	1	2	12,3	8,31	16,18	50	100,00%
348	Machetá (Cundinamarca)	7	6	1	11,56	19,25	3,4	600	100,00%
503	Guayatá (Boyacá)	3	2	1	9,64	12,81	6,45	200	100,00%
823	Chivor (Boyacá)	1	0	1	5,99	-	12,9	-	

Calculations of the rates per 10,000 inhabitants.

The geographical analysis performed considering beyond just the numerical value of the variable of interest (general rate), considering the proximity between points, finding areas of concentration of centenarians in the Valle de Tenza area, the Southern Antioquia and Northern Gran Caldas with La Celia and Ciudad Bolívar, and the Caribbean area with Sabana de Bolívar and the lower Magdalena River basin where the municipality of Piñon is located.

## Discussion

This study aimed to characterize individuals over the age of 95 in Colombia from a Geohealth perspective and contextualize these findings within the framework of previous research. The results underscore the importance of understanding the geographical distribution of the elderly population as a crucial component for developing public policies and programs aimed at improving their quality of life. In line with the NU-CEPAL (2022) report, which identified geographical, environmental, and cultural factors influencing longevity in Latin America and the Caribbean [28], our findings highlight specific areas in Colombia with a notable concentration of centenarians. This observation not only supports previous studies but also suggests the need for further research to identify the specific characteristics of these regions that may foster successful aging.

By approaching aging from a territorial perspective, as suggested by Sarabia C. (2009) and other authors, this study contributes to understanding the geographic clustering and its relationship with the health of the elderly population [29]. Territorial analysis is essential, as successful aging cannot be fully understood solely within the confines of traditional political-administrative boundaries; rather, it is influenced by local factors such as the physical environment and community support networks. This view aligns with the concept of "Blue Zones" identified by Poulain et al. (2004, 2013) and Buettner (2012), which emphasize the importance of environmental and lifestyle factors in promoting longevity [30, 31].

A critical component of this research was examining the rates, which allowed the identification of zones with a concentration of centenarians, rather than focusing solely on the frequency of individuals over 95 years old, as suggested by Rosselli et al. (2017) and Zapata-Bravo et al. (2024). In this regard, the most significant finding was the remarkable concentration of individuals over 95 years old in the Valle de Tenza region, which exhibits rates notably higher than the national average, [23, 32]. Additionally, it was found that between 75% and 100% of these individuals have resided in these municipalities for the past five years, suggesting the possible influence of territorial factors on longevity. Similarly, the frequency of centenarians was observed in densely populated urban areas such as Bogotá, Medellín, Cali, Barranquilla, and Cartagena. This pattern raises questions about the specific determinants of longevity in these rural and urban settings, consistent with previous research by Otero-Ortega A et al. (2020) and UN-HABITAT (2020), which explore the relationship between rural and urban growth, sustainable development, and health [33, 34]. Longevity in these regions and cities suggests that socioeconomic, environmental, and possibly genetic factors play a key role, although the interaction of these factors in the Colombian context remains to be clarified.

Additionally, the predominance of women among those over 95 years of age reflects previous studies documenting higher female life expectancy compared to men. From a sociodemographic perspective, data reveals that 63% of centenarians are women and 37% are men, reflecting a global trend previously documented in studies such as those by Ospina M (DANE, 2024) and Sandín-Vázquez M et al. (2008), which highlight the longer female life expectancy. This suggests the need for further research on the gender implications of longevity and well-being in older adults, particularly in the context of public health policies and programs [35, 36].

When comparing the results of this research with others, such as those by Azin et al. (2001) and Aliberti SM et al. (2024), in longevity zones such as Georgia and Cilento, similarities and differences can be observed regarding their geographical and ecological characteristics. In Georgia, particularly in the Caucasus Mountains, longevity has been associated with altitude and a temperate climate, conditions that favor an active lifestyle and a diet based on local products, such as fermented dairy. Similarly, in Cilento, a coastal mountainous region in southern Italy, longevity has been linked to a Mediterranean environment promoting a diet rich in vegetables, olive oil, and fish, alongside strong social cohesion, culture, spirituality, and a physically active life [37, 38]. In contrast, our study in Valle de Tenza reveals longevity influenced by similar factors such as diet and lifestyle, but in a different geographical and ecological context. Here, the mountainous topography, temperate climate, and fertile soils play an essential role in agricultural practices and access to fresh food, contrasting with the coastal areas of Cilento and the higher altitudes of Georgia. This comparative analysis reinforces the hypothesis that the interaction of geographical, ecological, and cultural factors, as partly mentioned by Gulis et al. (2000), is key to understanding life expectancy and longevity in different contexts, highlighting both common and unique factors contributing to healthy aging in each region [39].

However, it is important to address certain limitations of this study. First, the use of secondary data from the 2018 Census may not fully capture the most recent demographic changes, particularly following the COVID-19 pandemic, which has significantly impacted the elderly population worldwide. Moreover, being a cross-sectional and ecological study, it is not possible to follow the individual trajectories of centenarians over time, limiting our understanding of aging pathways in these groups.

Given the above, future research should focus on developing longitudinal studies that track the health and well-being of centenarians in both urban and rural areas. Such studies would provide a more detailed understanding of the factors promoting healthy longevity in specific contexts. Additionally, a broader approach considering additional sociodemographic variables, such as educational level, access to healthcare services, and family, social, spiritual, and community support networks, would be crucial to gain a more comprehensive understanding of longevity in Colombia.

The findings of this study not only contribute to the literature on aging in Colombia but also provide a framework for developing public policies aimed at improving the quality of life of the elderly. By identifying areas with high concentrations of centenarians, this research underscores the importance of contextual factors in longevity and opens the door to new studies that will help unravel the key factors of healthy aging in the country.

## Conclusion

This study has provided a detailed view of the geographical distribution of centenarians in Colombia, identifying significant concentrations in specific regions such as Valle de Tenza, south of Antioquia and north of Risaralda, Caribbean area—Bajo Magdalena and municipality of Piñón. These findings underscore the importance of considering both individual and contextual factors to understand longevity.

The results highlight the imperative need to adopt a comprehensive and territorial approach that contemplates the dynamic interaction between the organism, the environment, and socioeconomic and cultural circumstances. This holistic perspective is crucial to understanding successful aging and goes beyond the traditional pathological approach that has dominated until now.

By examining contextual factors, such as geographic and social conditions that support longevity, more effective strategies can be developed to improve quality of life for older populations. The identification of areas with high concentrations of centenarians suggests the need to implement specific policies and programs that address the unique needs of these populations in terms of health and well-being.

This multidimensional approach will facilitate more effective formulation of policies and programs aimed at supporting older people and promoting healthy aging. The study findings also have significant implications for future research, especially in exploring the specific determinants that contribute to longevity in different geographic and cultural contexts within Colombia.

Understanding the factors that promote a long and healthy life will allow the development of more effective and personalized interventions that benefit older populations. The identification of these longevity "clusters" provides a solid basis for the implementation of strategies aimed at improving the quality of life and well-being of the elderly in Colombia.

## Supporting information

S1 Table Censo 2018. (XLSX)
